# Secondary Students' Writing Achievement Goals: Assessing the Mediating Effects of Mastery and Performance Goals on Writing Self-Efficacy, Affect, and Writing Achievement

**DOI:** 10.3389/fpsyg.2017.01406

**Published:** 2017-08-21

**Authors:** Meryem Yilmaz Soylu, Mary G. Zeleny, Ruomeng Zhao, Roger H. Bruning, Michael S. Dempsey, Douglas F. Kauffman

**Affiliations:** ^1^Independent Researcher Ankara, Turkey; ^2^Department of Educational Psychology, University of Nebraska-Lincoln Lincoln, NE, United States; ^3^Department of Surgery, Boston University School of Medicine Boston, MA, United States

**Keywords:** achievement goals, writing, secondary school children, self-efficacy, affect, achievement

## Abstract

The two studies reported here explored the factor structure of the newly constructed Writing Achievement Goal Scale (WAGS), and examined relationships among secondary students' writing achievement goals, writing self-efficacy, affect for writing, and writing achievement. In the first study, 697 middle school students completed the WAGS. A confirmatory factor analysis revealed a good fit for this data with a three-factor model that corresponds with mastery, performance approach, and performance avoidance goals. The results of Study 1 were an indication for the researchers to move forward with Study 2, which included 563 high school students. The secondary students completed the WAGS, as well as the Self-efficacy for Writing Scale, and the Liking Writing Scale. Students also self-reported grades for writing and for language arts courses. Approximately 6 weeks later, students completed a statewide writing assessment. We tested a theoretical model representing relationships among Study 2 variables using structural equation modeling including students' responses to the study scales and students' scores on the statewide assessment. Results from Study 2 revealed a good fit between a model depicting proposed relationships among the constructs and the data. Findings are discussed relative to achievement goal theory and writing.

## Introduction

Achievement goal theory portrays human behavior as being goal directed, and suggests that individuals pursue goals in diverse ways within particular content domains and performance contexts (Nicholls et al., 1990; Dweck, 2000; Linnenbrink and Pintrich, 2002; Elliot et al., 2011). In academic settings, achievement goals influence learners in how, or if, they engage learning tasks (Pintrich, 2003; Elliot et al., 2010). Learners' self-efficacy for goal-relevant tasks influences, and is influenced by, the achievement goals that frame those tasks (Pajares et al., 2000; Pekrun, 2006; Hadwin and Webster, 2013). Self-efficacy beliefs, furthermore, often have an affective valence that can influence learners' goal pursuits (Linnenbrink and Pintrich, 2002; Pekrun, 2006; Pekrun et al., 2006).

Writing research emphasizes composing as a goal-directed process (Hayes and Flower, 1980; Hayes, 1996; Harris et al., 2009; Fayol, 2012; MacArthur et al., 2016). Writing goals are seen as hierarchical. High-level goals (e.g., establishing a purpose and an audience for the text) are supported by sub-goals (e.g., identifying cogent arguments needed for persuasion). Writing goals are also recursive: goals and sub-goals assert and reassert themselves throughout the writing process as new challenges arise from the text being produced. Writing research has further shown a consistent relationship between self-efficacy for writing and writing performance. A given writer's self-efficacy may vary widely across specific domains (e.g., writing a science report for biology class as compared with creative writing in an English class) and also for a specific task performed in different contexts (e.g., writing a term paper for class as compared with writing an article for publication in a professional journal) (Bruning and Kauffman, 2015). Numerous studies support the common experience that writing is a taxing cognitive activity that can engender frustration and negative affect (Zimmerman and Kitsantas, 1999; Pajares et al., 2000; Pajares and Cheong, 2003; Boscolo, 2009).

Relatively few studies have explored the role of achievement goals in the writing process, however. In one exception, Kaplan et al. (2009) studied self-regulation and writing and found that some students' writing strategies are located within an achievement goal framework. Their study, however, did not explicitly test the role of achievement goals in writing, and to our knowledge, such an explicit test has not yet been conducted. The purpose of the present studies, therefore, was to test whether students' writing-related behaviors fit a *trichotomous* achievement goal framework, and whether these achievement goals are related to students' writing attitudes and performance.

### Achievement goal research

McClelland, Atkinson, and their colleagues have helped establish achievement theory as an important focus of motivation research (e.g., McClelland, 1953/2015). Researchers continue to advance the field under one of two broad headings: *goal theory* and *goal orientation*. Goal theory addresses the role *purpose* plays in human motivation (Anderman and Maehr, 1994; Urdan and Maehr, 1995; Eccles and Wigfield, 2002). To improve students' achievement, these studies suggest, instruction should help them set specific and challenging goals and provide them with accurate and relevant progress-related feedback (e.g., Schunk and Swartz, 1993; Locke and Latham, 2002). Instituting these measures, researchers argue, can increase students' performance, build their self-efficacy for writing, and encourage them to set more challenging goals. Recent studies by Hadwin and colleagues (Hadwin et al., 2011; Hadwin and Webster, 2013) further suggest that students' self-regulation improves over time with instructional supports designed to complement their classroom goals.

Goal orientation research, in contrast, focuses on the *reasons* individuals have for pursuing goals. Nicholls et al. (1990) have suggested a two-dimensional goal orientation model in which learners are guided by either a *task* or an *ego* goal. Task-involved students show higher intrinsic motivation and are less anxious about failure. Ego-involved students, in contrast, are more likely to engage in tasks that can confirm their self-concept. Failure to perform ego-tasks successfully can challenge self-concept and elicit anxiety, however. Dweck and Leggett (1988) conceptualized this two-dimension model somewhat differently. They argued that learners' approach goals derived from either a *mastery orientation* or a *performance orientation*. Mastery-oriented students focus on learning for its own sake, on attaining deep understanding, and for their self-improvement. Performance-oriented students, however, tend to pursue goals with the intent of maximizing their perceived competence and avoiding the appearance of incompetence. More recent research has shown, however, that performance-oriented goals can be adaptive, maladaptive, or have no effect on students' performance (Linnenbrink and Pintrich, 2002; Linnenbrink, 2005).

Elliot and colleagues have further argued that performance-oriented individuals specifically endorse either an approach or an avoidance goal (Elliot and Harackiewicz, 1996; Elliot and Church, 1997). Learners, in this view, seek either to obtain an optimal outcome or to preclude a negative one. Students pursuing performance approach goals are more likely to engage in activities that offer a high probability for success. Successful performance may demonstrate personal aptitude and ability. Avoidance-oriented students, by contrast, seek to evade challenging goals, which they perceive as potential threats to their self-concept (Elliot and Covington, 2001). Taken together, performance approach, performance avoidance approach, and mastery orientation, have come to be called the *trichotomous* model of achievement goals.

Elliot and colleagues continued to refine the achievement goal model by extending the valence structure of performance goals to mastery goals resulting in mastery approach and mastery avoidance orientations, which together with the two performance orientation valences created a 2 × 2 model (Elliot, 1999; Elliot and McGregor, 2001). Elliot et al. (2011) further clarified the 2 × 2 model by introducing three *goal types*: tasks, self, and other. These goal types, when crossed with the competence-related valences of the 2 × 2 model, result in a 3 × 2 model. In this model, mastery goals are posited as those one might achieve through competent performance (task goal), by striving to further one's own mastery of learning (self goal), or by engaging in deep learning (other goal). The 3 × 2 model thus represents six separate goal-related constructs. These more recent iterations of the achievement goal model have been well received generally, although none has as yet achieved the wide adoption the trichotomous model has enjoyed. Thus, the present study is framed within the trichotomous framework.

Achievement goal theory also has undergone two other notable refinements. First, empirical findings support a *multiple goal perspective*, where under certain conditions performance approach goals prove adaptive. When combined with mastery goals, the additional goals appear to boost learners' motivation overall (Linnenbrink, 2005; Senko et al., 2011). Second, Elliot and Covington (2001) and Pekrun (2006) have argued that individuals' achievement goals are content and context specific. From this perspective, a student may endorse mastery goals in one course, performance approach goals in another, and performance avoidance goals in a third.

### Writing, achievement goals, self-efficacy, performance, and affect

Research has generally shown that achievement goals predict individuals' motivation, self-beliefs, and performance. However, no other studies to date, to the knowledge of the research team, have explicitly explored the relationship of achievement goals to authentic writing performance, although there is strong empirical evidence supporting a relationship between achievement goals and writing self-efficacy (Bruning and Kauffman, 2015) in diverse academic settings and with different groups of learners (Pajares et al., 2000; Shim and Ryan, 2005). Students who endorse mastery goals generally demonstrate high self-efficacy, although the relationship between performance goals and self-efficacy is less clear (Pajares et al., 2000; Linnenbrink, 2005; Shim and Ryan, 2005; Kaplan et al., 2009). There is also evidence to support the mediating role of achievement goals and self-efficacy on academic performance. Bouffard et al. (2005), for example, found significant interactions between mastery goals and self-efficacy on writing achievement, though not between performance goals and self-efficacy. Other researchers have studied the broader relationship of achievement goals and affect, as well as how affect mediates achievement goals' and performance (Elliot and Church, 1997; Leach et al., 2003). The writing domain, then, likely shapes achievement goals and self-efficacy to mediate writing performance.

There is ample evidence to suggest that writing quality is associated with three aspects of the writing process: *syntactic knowledge, ideational fluency*, and *self-regulation* for writing (Bruning et al., 2013). *Syntactic knowledge* establishes coherence and cohesion in writing (Langacker, 1987, 2008; Evans and Green, 2006), supports higher-order writing processes, and by extension affects writing performance (Pajares, 2003, 2007; Halliday and Hasan, 2004; Halliday and Matthiessen, 2004; Myhill, 2010). *Ideational fluency* represents writers' ability to generate and articulate relevant content (Bruning et al., 2013), and relies on deep and broad connotative and denotative knowledge of lexical forms that are embedded in well-developed schematic structures (Langacker, 1987, 2008; Evans and Green, 2006). Because ideational fluency is central to writing performance, it affects writers' perceptions of their own writing competence (Hupet et al., 1998). *Self-regulation* gives writers control over the syntactic and semantic dimensions of writing (Zimmerman and Bandura, 1994; Zimmerman and Kitsantas, 2002; Bruning et al., 2013). Finally, self-regulation strategies assist writers in accessing the syntactic and semantic knowledge necessary for coordinating critical behaviors related to syntactic and semantic performance. Self-regulation, furthermore, supports evaluation of syntactic and semantic goals and helps writers adopt appropriate writing strategies and maintain focus and control over their emotions during the writing process (Bruning et al., 2013).

In addition to self-efficacy, individuals' affect for goal-related tasks can influence their motivation for engaging in and persisting with the writing process. Pekrun et al. (2006), for example, argued that affect profoundly influences writing achievement goals. Other researchers have studied the broader relationship of achievement goals and affect, as well as how affect mediates achievement goals' and performance. Significantly, Pekrun et al. (2006) found that different achievement goals predicted different types of emotions and that most of these emotions mediated the relations between achievement goals and performance.

### The present studies

Two coordinated studies were conducted as part of a large-scale assessment of elementary school, middle school, high school, and college students' motivation and beliefs about writing. The purpose of the first study was to provide initial validation of a new writing goals instrument, the Writing Achievement Goal Scale (WAGS). This study used the middle school data to examine the factor structure of students' responses using confirmatory factor analysis. The CFA offered initial validation of the scale to warrant moving forward with Study 2, which is described later.

Together we expected these studies to enhance our understanding of writing achievement goals in several ways. First, we sought to explore explicitly the relationship of achievement goals to writing performance and affect. Kaplan et al. (2009) included writing as a variable in their study of achievement goals, but they did not explore the role of achievement goals to performance. Second, we are not aware of any studies that have tested achievement goals and writing in an authentic academic context. Our studies utilized assessment data that was part of the normal assessment practices of the local school district, while Kaplan et al. (2009) conducted their study in an experimentally controlled environment. Testing writing achievement goal constructs in authentic settings has the potential to support the development of stronger and better-calibrated writing interventions. Finally, whereas Kaplan and colleagues assessed writing performance immediately after students completed an achievement scale, writing performance data in our studies was gathered by the local school district approximately 2 months later. Lagged data collection has the potential to more precisely explicate the relationship of goal resiliency and writing performance.

## Method for study 1

The purpose of Study 1 was to seek initial evidence for the reliability and validity of the Writing Achievement Goals Scale (WAGS). For this purpose, we opted to use the trichotomous achievement goal model because it is the one most widely utilized in K-12 research. The WAGS was constructed as part of the overall development of the Writing Habits and Beliefs Survey (WHBS) (Bruning et al., 2013). The WHBS comprises several scales related to goals, self-efficacy, affect, and performance. If Study 1 supported the reliability and validity of the WAGS, our plan was to next test relationships among the WAGS constructs and other motivational and outcome variables.

### Participants

Six hundred ninety-seven middle school students from four schools in a mid-size Midwestern US city completed the WHBS. The sample included all students enrolled in 8th grade English/Language Arts classes at the four schools and was representative of the 8th grade student body in the entire school district. Of those reporting gender (*n* = 690), 325 were boys and 365 were girls. Mean reported age (*n* = 676) was 13.8 years. Of the students reporting their grade level (*n* = 692), 690 (98.6%) were 8th graders, one was a 7th grader, and one was a 9th grader. In terms of ethnicity (*n* = 681), 420 students self-reported themselves as Caucasian, 66 as African-American, 53 as Latino/Latina, 36 as Asian/Pacific Islander, and the remaining 106 students identified themselves as either multiracial or other ethnicity. Finally, of the 690 students reporting on the primary language spoken at home, 580 (approximately 84%) of the participants indicated English was the primary language spoken at home, while the remaining 110 students reported languages other than English were spoken at home. These statistics are consistent with school district demographics.

### Measures

#### Writing achievement goals scale (WAGS)

Students' writing achievement goals were assessed with the WAGS, which was designed by the research team to assess students' intentions for writing. Students responded to items on a 5-point Likert-type scale ranging from 0 (*does not describe me at all*) to 4 (*describes me perfectly*). The researchers reviewed existing achievement goal scales to help generate items suitable for each subscale (Miller et al., 1996; Midgley et al., 1998; Urdan and Midgley, 2003; Elliot and Murayama, 2008). During scale development, we also adhered to recommendations for constructing achievement goal scales suggested by Elliot and Murayama (2008). First, items were written using precise wording that focused on *commitment* to a future behavior and not on a *value* placed on that behavior. Each item was anchored with the stem, “When I'm writing in my English/Language Arts class, I am trying to…” Second, items were written so as to separate the *rationale* for the goal from the *goal itself*. Specifically, we generated items intended to assess students' *intention* rather than the anticipated *outcome*. Our items were developed, furthermore, to avoid implicating non-goal variables such as emotions, preference, or concern. Third, we worked to avoid our own potential bias for one goal over another. According to Elliot and Murayama (2008), this practice is particularly important because goals should correlate with one another and students likely possess multiple goals for the same task. Consequently, we avoided phrases such as, “I just want to pass…” or “My only goal is to understand…” Instead, we wrote items that included phrases such as, “When I'm writing in my English/Language Arts class, I'm trying to improve how I express my ideas.” The final 12-item version of the questionnaire included three subscales (4 items each) assessing individuals' orientation for mastery, performance approach, and performance avoidance goals (See Table 1).

**Table 1:** Final model parameters for a three-factor model of writing achievement goals in Study 1.

	**Factor loadings**	**Standard error**	**Standardized values**
**When I'm writing in my English/Language arts class, I'm trying to …**			
**Mastery Goals**			
1. Become a better writer.	1.000	0.000	0.799
2. Learn to choose words that best express my ideas.	0.978	0.041	0.823
3. Improve how I express my ideas.	1.018	0.041	0.838
4. Better organize my ideas.	0.991	0.043	0.800
**Performance Approach Goals**			
5. Impress my teacher with my writing.	1.000	0.000	0.636
6. Be a better writer than my classmates.	0.920	0.058	0.728
7. Show off my writing skills.	1.281	0.073	0.835
8. Be the best writer in my class.	1.381	0.077	0.869
**Performance Avoidance Goals**			
9. Hide that I have a hard time writing.	1.000	0.000	0.803
10. Keep people from thinking I'm a poor writer.	0.984	0.042	0.806
11. Keep my teacher from thinking I'm not very smart.	0.964	0.041	0.816
12. Avoid looking foolish in front of my classmates.	0.803	0.039	0.734

### Procedures

Permission to conduct this study was obtained from the university's institutional review board (IRB), from the participating schools' district office, from the principals at the participating middle schools, and from the English/Language Arts teachers at the four participating schools. A letter informing parents/guardians about the study was distributed to the participating English/Language Arts teachers and sent home with the students. The letter explained that students would be taking a writing survey that would take approximately 20 min to complete, that results were confidential, and that participation in the study was voluntary and would have no effect on the students' grades or relationships with teachers or the school. All students confirmed voluntary participation. Teachers administered the survey at the start of the students' eighth grade English/Language Arts classes. Completed surveys with identifiers were returned to the district office.

## Results for study 1

For the purposes of Study 1 we report only results pertaining to the Writing Achievement Goals Scale. Study 1 was designed to determine whether the WAGS data better fit the traditional mastery and performance orientation model or the hypothesized three-factor model including mastery, performance approach, and performance avoidance goals. We performed a confirmatory factor analysis (CFA) using MPlus v. 7.0 (Muthén and Muthén, 2012) and judged the corresponding model fits against the standard criteria recommended by Hu and Bentler (1999). We chose CFA over EFA for two reasons. First, the models we tested represent two well-established theoretical models of achievement goals, namely the mastery-performance goal model and the three-factor model that includes mastery, performance approach, and performance avoidance goals. Second we began our investigation with a preformed theoretical outcome in mind.

We first tested how well student responses fit a traditional two-factor achievement model corresponding to mastery and performance goals. The resulting fit was poor, χ^2^_(53)_ = 674.95, *p* < 0.001, CFI = 0.776, TLI = 0.725, RMSEA = 0.131, SRMR = 0.098. Next, we assessed how well the data fit the hypothesized three-factor model corresponding to mastery, performance approach, and performance avoidance goals. Results of this analysis indicated a good fit for the data and the model, χ^2^_(51)_ = 182.88, *p* < 0.001, CFI = 0.952, TLI = 0.909, RMSEA = 0.061, SRMR = 0.047. Finally, in order to determine whether the data fit the three-factor model significantly better than it did the two-factor model, we conducted a chi square difference test. Results of this analysis were significant, χ^2^ difference (2) = 492.072, *p* < 0.001. Because the survey items fit the model conceptually as well as statistically, no further analyses were conducted (See Table 1 for factor loadings). We also performed normality tests on the three writing achievement goals and the results indicated the trichotomous variables did not follow a normal distribution (Learning Goals: Shapiro-Wilk statistics = 0.957, *p* < 0.001; Performance Approach Goals: Shapiro-Wilk statistics = 0.973, *p* < 0.001; Performance Approach Goals: Shapiro-Wilk statistics = 0.952, *p* < 0.001). No outliers were observed that might undermine the potential normal distribution.

Means, standard deviations, and correlations for the three writing achievement goals are presented in Table 2. Consistent with current achievement goal theory (e.g., Elliot and Murayama, 2008), the three scales showed significant positive correlations with one another. The correlation between mastery goals and performance approach goals was the strongest (*r* = 0.613), while performance approach and performance avoidance goals had a moderate correlation (*r* = 0.366). The smallest observed correlation was between mastery goals and performance avoidance goals (*r* = 0.211). Internal consistencies for each of the subscales were moderate but acceptable. Alpha for the mastery goals items was 0.843, for performance approach items 0.806, and for the performance avoidance goal items 0.751.

**Table 2:** Means, standard deviations, and correlations of the three writing achievement goals in Study 1.

**Variable**	***M***	***SD***	**1**	**2**	**3**
1 MG	2.307	0.924	1.000		
2 PApG	1.784	1.033	0.661^**^	1.000	
3 PAvG	1.864	0.931	0.306^**^	0.380^**^	1.000

*MG, mastery goals; PApG, performance approach goals; PAvG, performance avoidance goals*.

** *Correlation is significant at the 0.01 level*.

Study 1 demonstrated sufficient internal consistency for the WAGS in the middle school sample, with the internal consistency for each factor also consistent with reliability estimates of other achievement goal scales. These findings suggested that the research team could move on to the next phase of analysis in Study 2.

## Method for study 2

The purpose of Study 2 was to explore relationships between writing achievement goals, self-efficacy, affect, and performance. For this purpose, we used utilized response data to several scales comprising WHBS, including the WAGS, Self-Efficacy for Writing Scale (SEWS) (Bruning et al., 2013), Liking Writing Scale (LWS) (Kauffman et al., 2010), and students' scores on the statewide writing assessment.

### Participants

Participants included 572 high school students enrolled in 11th grade English/ Language Arts classes from two public high schools in a mid-sized Midwestern city. To insure continuity between Study 1 and Study 2 groups, we previously had collected data at four middle schools that were feeder schools for the two high schools participating in Study 2. The majority of participants (*n* = 520) were 11th graders and 37 were 12th graders. There were 292 males in the sample and 261 females. Participants' average age was 16.76 (*SD* = 0.77), with a modal age of 17. Of the 553 students reporting ethnicity, 390 students identified themselves as Caucasian, 37 identified themselves as African-American, 31 as Latino/Latina, 31 as Asian/Pacific Islander, and 64 as either multiracial or other ethnicity. A majority of the students (*n* = 480) reported English as the primary language spoken at home, while 75 students reported other languages were primarily spoken there. Secondary school students' demographics mirrored those of the middle school sample in Study 1. Four hundred-seventy matches were made between secondary students and scores from the statewide writing assessment.

### Measures

Materials included measures assessing students' writing achievement goals, how well they like writing, writing self-efficacy, their self-reported writing grades, and scores from the statewide writing assessment that we were able to match to our sample.

#### Writing achievement goals

Students' writing achievement goals were assessed with the same 12-item the WAGS used in Study 1.

#### Writing affect

Students' feelings about writing were measured with the Liking Writing Scale (LWS) (Bruning et al., 2013). The LWS consists of four items asking students to rate their overall feelings (positive and negative) about writing. Students responded on a 5-point Likert-type scale ranging from 1 (*strongly disagree*) to 5 (*strongly agree*). This scale assessed how students felt about writing, including such items as “I feel happy when I write” and “I get anxious when I have to write” (reverse coded). Item analysis showed good reliability for this scale (α = 0.84).

#### Writing self-efficacy

Students' writing self-efficacy was measured using the Self-Efficacy for Writing Scale (SEWS) (Bruning et al., 2013). SEWS consists of 16 items representing three writing self-efficacy subscales. *Self-efficacy for conventions* items asked students to rate the probability they could successfully use grammatical and syntactic rules (α = 0.86), for example, “I can punctuate my sentences correctly.” *Self-efficacy for ideation* items asked students to rate the probability they could generate sufficient ideas for their writing (α = 0.92), for example, “I can put my ideas into writing.” *Self-regulation self-efficacy* items asked students to rate the probability they could coordinate behaviors specific to syntactic and semantic fluency (α = 0.91), for example, “I can avoid distractions when I write.”

#### Statewide writing assessment (SWA)

The Statewide Writing Assessment was a comprehensive 2-day performance assessment given to all 4th, 8th, and 11th grade students in the Midwestern state where the research was conducted (Dappen et al., 2008). At the 11th grade level, the writing prompt asked students to generate a persuasive essay about a specified topic over a 2-day period in which students received the prompt and spent time planning their responses on the first day, and completed their writing in an untimed session on the second. All essays generated during the statewide writing assessment were hand scored by two teachers with grade-level expertise in student writing. The two raters assigned each essay a score on a 1–4 scale, and the two scores were combined, resulting in a global score ranging from 2 to 8. According to the state's Department of Education, interrater reliabilities for the statewide writing assessment were generally high (97% exact or adjacent agreement reported for the 2007 assessment cycle). Finally, the participating school district's research and evaluation team used a blinded process to match the secondary school students' SWA scores to their responses to the WHBS. The data were then forwarded to the research team through another blinded process.

### Procedure

The research team first obtained permission to conduct the study from the university's Institutional Review Board. Next, the team sought and received permission to conduct the study from the school district's research compliance office as well as from all principals and English/Language Arts teachers at the participating schools. A letter explaining the project was sent home to students' parents/guardians. No students opted out. As with Study 1, students completed the WHBS during the first 20 min of English/Language Arts class approximately 6 weeks prior to the statewide writing assessment.

## Results for study 2

### Preliminary analyses

Means, standard deviations, and correlations for each variable are shown in Table 3. As can be seen, students reported higher mastery goals (*M* = 2.534) than for either performance approach goals (*M* = 1.672) or performance avoidance goals (*M* = 1.197). Of the three self-efficacy dimensions, students reported highest confidence for writing conventions (*M* = 84.387). Self-regulation for writing was rated lowest (*M* = 62.625). Students also reported somewhat positive affect toward writing (*M* = 3.433). Both mastery goals and performance-approach goals correlated positively with self-efficacy for writing, affect toward writing, and writing performance. Mastery goals correlated higher with writing performance than with performance approach goals. (Note: It is interesting that the correlations among the three dimensions of self-efficacy were relatively high, indicating that some of these dimensions might be predicting the others.) Finally, affect toward writing correlated more highly with mastery goals than with writing self-efficacy.

**Table 3:** Means, standard deviations, and correlations among variables tested in Study 2.

**Variable**	***M***	***SD***	**1**	**2**	**3**	**4**	**5**	**6**	**7**
1 MG	2.534	1.008	1.000						
2 PApG	1.672	1.049	0.570^**^	1.000					
3 PAvG	1.197	0.986	0.230^**^	0.381^**^	1.000				
4 SE-C	84.387	14.428	0.210^**^	0.237^**^	−0.114^**^	1.000			
5 SE-I	73.565	18.987	0.397^**^	0.368^**^	−0.065	0.530^**^	1.000		
6 SE-SR	62.625	23.017	0.451^**^	0.368^**^	−0.078	0.440^**^	0.707^**^	1.000	
7 LWS	3.433	0.925	0.515^**^	0.314^**^	−0.102^*^	0.225^**^	0.487^**^	0.497^**^	1.000
8 SWA	6.083	1.288	0.213^**^	0.194^**^	0.026	0.378^**^	0.203^**^	0.206^**^	0.133^**^

*MG, mastery goals; PApG, performance approach goals; PAvG, performance avoidance goals; SE-C, self-efficacy for conventions; SE-I, self-efficacy for ideation; SE-SR, self-efficacy for self-regulation; LWS, Liking Writing Scale; SWA, statewide writing assessment*.

* = p ≤ 0.05;

** *= p ≤ 0.01*.

Normality tests were performed on all variables in the model, and none of the variables followed a normal distribution. A few outliers were observed for self-efficacy for conventions and ideation as well as for the State Writing Assessment scores. After excluding outliers, these variables did not follow a normal distribution, and based on this result, we felt confident in moving forward with the CFA. CFA using maximum likelihood method is quite robust to the deviation from normality.

### CFA results

We again employed a confirmatory factor analysis (CFA) using MPlus to test the fit of the variables to the three-factor model. As stated previously, we chose CFA over EFA because our model testing was based on theoretically supported relationships among the variables. CFA is appropriate when testing a model for with a preexisting theoretical model, as we were doing. We used maximum likelihood estimation. Results revealed an acceptable fit between the data and the model, χ^2^_(51)_ = 276.70, *p* < 0.001, CFI = 0.930, RMSEA = 0.089, SRMR = 0.064 using evaluation criteria established by Hu and Bentler (1999). We conducted no further analyses because the fit for the secondary school data was similar to the fit for the middle school data and results were conceptually and theoretically strong. Factor loadings for the high school sample are presented in Table 4. We next assessed the internal consistency of each of the three factors. Mastery achievement goals showed the highest internal consistency (*r* = 0.86), followed by performance approach (*r* = 0.83), and performance avoidance (*r* = 0.78). The three achievement goals correlated differentially, which is consistent with achievement goals research (Elliot and Murayama, 2008) and with results from Study 1. Specifically, the correlation between mastery goals and performance approach goals was highest at 0.57, followed by performance approach with performance avoidance goals (*r* = 0.38), and mastery with performance avoidance goals (*r* = 0.23) (See Table 3).

**Table 4:** Factor loadings for the three-factor WAGS model in Study 2.

	**Factor loadings**	**Standard error**	**Standardized values**
**When I'm writing in my English/Language arts class, I'm trying to …**			
**Mastery Goals**			
1. Become a better writer.	1.000	0.000	0.662
2. Learn to choose words that best express my ideas.	1.121	0.074	0.732
3. Improve how I express my ideas.	1.384	0.080	0.896
4. Better organize my ideas.	1.326	0.077	0.851
**Performance Approach Goals**			
5. Impress my teacher with my writing.	1.000	0.000	0.632
6. Be a better writer than my classmates.	1.232	0.088	0.764
7. Show off my writing skills.	1.308	0.089	0.782
8. Be the best writer in my class.	1.373	0.095	0.824
**Performance Avoidance Goals**			
9. Hide that I have a hard time writing.	1.000	0.000	0.414
10. Keep people from thinking I'm a poor writer.	2.620	0.285	0.845
11. Keep my teacher from thinking I'm not very smart.	2.349	0.253	0.788
12. Avoid looking foolish in front of my classmates.	2.229	0.249	0.705

### Path analysis model evaluation

**Figure 1:**
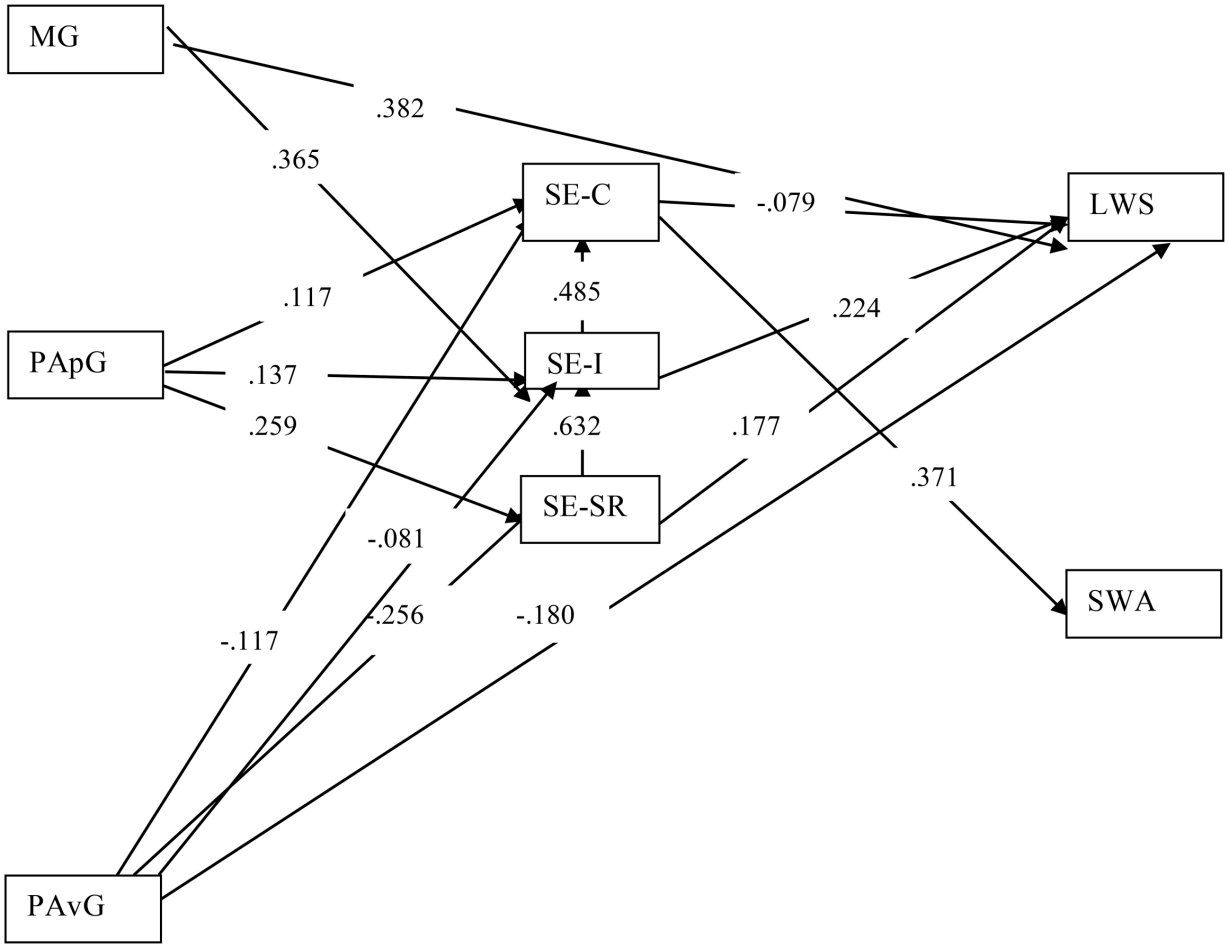
Tested model of the relationships among achievement goals, self-efficacy, affect, and writing performance in Study 2. MG, mastery goals; PApG, performance approach goals; PAvG, performance avoidance goals; LWS, Liking Writing Scale; SE-C, self-efficacy for convention; SE-I, self-efficacy for ideation; SE-SR, self-efficacy for self-regulation; SWA, score on the statewide writing assessment.

We tested the model using structural equation modeling and MPlusv.7.0 (Muthén and Muthén, 2012). We added two paths to our original hypothesized model to account for relatively high correlations among the three dimensions of self-efficacy. Maximum likelihood was the method of estimation. Loading coefficients were considered salient if they were above 0.25 or 0.30. Once the additional paths were added the data had good fit with the model (*N* = 561): χ^2^ = 4.296, *df* = 1, *p* = 0.0382; CFI = 0.997; TLI = 0.927; RMSEA = 0.077; SRMR = 0.008, and the SRMR dropped significantly from above the 0.05 threshold to well below it after adding the additional paths. The tested model explained 33.3% of the variance in self-efficacy for conventions, 55.8% of the variance in self-efficacy for ideation, 30.2% of the variance in self-efficacy for self-regulation, 40.5% of the variance in liking writing, and 16.9% of the variance in Statewide Writing Assessment scores. Figure 1 shows relationships among variables in the new model. Significant paths are presented with their standardized coefficients.

## Discussion

As described earlier, Studies 1 and 2 formed a tandem research design. The purpose of Study 1 was to conduct an initial validation test for the newly constructed WAGS. Results from Study 1 indicated that a more thorough testing of the scale's reliability and validity was warranted. Study 2 represents the capstone of the two studies and is the focus of our discussion here. It had two purposes. First, we were interested in learning if the WAGS was replicable, which we tested with data from both middle and high schools. Our analysis revealed that the high school student responses were consistent with middle school student responses and with a three-factor achievement goals model. Analysis also indicated that internal consistencies for the three factors in the high school group were similar to the middle school sample. Mastery items were found to possess relatively high internal consistency for both middle and high school students (*r* = 0.84 and *r* = 0.86, respectively), as did the performance approach items (*r* = 0.81 and 0.83 respectively). Internal consistencies for performance avoidance items, although more modest in magnitude, were also similar for the two groups (*r* = 0.75 and *r* = 0.78, respectively). Looking at the fit statistics, results for the high school data did not fit the three-factor model as well as the middle school data, but the fit was still within the acceptable range and thus offer initial evidence for the validity and reliability of the WAGS.

Our second purpose in Study 2 was to examine how well the 11th grade data fit our hypothesized theoretical model. Figure 1 shows how achievement goals differentially predict self-efficacy for writing, liking writing, and writing achievement. Overall, the three achievement goals were quite different in how they predicted—or did not predict—writing achievement or affect for writing. None of the achievement goals showed a direct relationship to writing achievement. Approach goals showed a positive indirect relationship with writing achievement by way of self-efficacy for conventions, as was the case for performance avoidance goals. These findings suggest two possible interpretations. First, the lack of significance might be interpreted as evidence that the relationship between writing achievement goals and writing performance is unsubstantial. There is ample evidence in the literature, however, to reject this interpretation (e.g., Kaplan et al., 2009), and we think it more likely that these results are related to contextual and temporal factors associated with the design of our study.

A second interpretation takes into account contextual factors surrounding the WHBS and the statewide writing assessment. It is possible that students interpret classroom writing differently from writing for high stakes assessments and thus have different goals for the two tasks. In the present study, we asked students to describe what they do when they write in their English/Language Arts classes, but related these responses to scores on the compulsory statewide writing assessment. In general, it might be argued that classroom writing is more closely linked to students' mastery and performance goals, while a statewide writing assessment may represent a writing task students feel compelled to complete for distant and abstract purposes. Thus, it may be that student responses to the WHBS in our study may not have reflected how they would have responded had we queried them on their writing achievement goals for the statewide assessment. Writing purpose creates an important—though not always positive—context for students' achievement goals. For example, it is possible that students pursue different sets of goals for writing in general, for particular writing genres and courses, and for specific writing assignments, suggesting that writing achievement goal research would benefit from careful identification of the kinds of writing goals and contexts being assessed.

Mastery goals and performance avoidance goals had a direct relationship to “liking writing,” a variable provided by a measure designed to tap students' motivational affect for the task. Performance approach goals, however, did not show a direct effect on affect for writing. Among possible reasons for these findings are that: (1) students with mastery goals may view the processes and products of writing itself as rewarding and thus enjoy engaging in writing activities; (2) students with performance avoidance goals are more likely to possess feelings of low competence and anticipate failure; and (3) students endorsing performance approach goals may view writing outcomes as more important than the writing process, that is, mainly as a means to an end and thus less relevant to affective experiences. These findings suggest the importance of attending closely to the affective components of writing in future interventions.

The relationship between achievement goals and the self-efficacy subscales differed in strength and direction. Performance approach goals were positively related to the three self-efficacy measures and were most closely related to self-efficacy for self-regulation. The same pattern was found for performance avoidance goals, but here the relationships were all negative. We would suggest students seeking performance goals—in contrast to those seeking mastery goals—may be more concerned with maximizing desirable performance or minimizing poor performance. As a result, individuals endorsing either of the performance goals may focus on the task itself, striving to meet domain-specific task requirements more closely linked to conventions and ideation. Mastery goals, in contrast, showed a strong relationship with self-efficacy for self-regulation, but not to either self-efficacy for conventions or ideation. Students with mastery goals may focus on engagement rather than on maximizing desirable or minimizing undesirable performance, suggesting that the behavioral outcomes of writers who endorse mastery goals relies more on self-regulatory behaviors than on either writing conventions or idea generation.

In conclusion, we highlight two findings from this study that we feel underscore the importance of the interplay between the dimensions of writing self-efficacy, as well as the need to further investigate the role achievement goals play in writing motivation. First, achievement goals differentially related to self-efficacy dimensions, with mastery goals predicting only self-efficacy for self-regulation, while the two performance goals predicted all three dimensions of self-efficacy. At the same time, the three goal types more strongly predicted self-efficacy for self-regulation than the two other self-efficacy dimensions. Second, results from the present studies indicate that all the self-efficacy dimensions correlated with liking writing, but only self-efficacy for conventions correlated with writing performance.

### Limitations and future directions

The findings from the present studies extend our understanding of the complex relationships between writing achievement goals, self-efficacy, affect, and writing performance. We discuss here, however, four important limitations to these studies that may have moderated the relationships between writing achievement goals and the other variables, most notably writing performance. As noted above, in our surveys we queried students about their goals for writing in their English/language arts classes, but the measure for performance was a statewide writing assessment, which they may perceive as a task external to the classroom environment. Future research should insure that writing tasks are clearly connected to the instruments being used to assess writing goals and motivations. As we move forward, we expect to address this issue more closely using our writing achievement goals and self-efficacy surveys in relationship to classroom writing contexts.

Second, this study did not assess indirect effects of writing achievement goals on writing performance. Our results indicate, for example, that performance approach goals had a direct effect on self-efficacy for writing conventions, which in turn had a direct effect on writing achievement. It is likely, however, that achievement goals also have an indirect effect on writing achievement through self-efficacy, affect, and other psychological and environmental factors. We chose not to test indirect effects for our model in the current study because our purpose was to validate the WAGS. We concluded, therefore, that an assessment of indirect effects was outside the scope of this validation study. We intend to study indirect paths in this model in future, however, because we think they play an important role in explaining writing achievement.

A third limitation relates to the wording of items in the WAGS. The middle school and high school students may have interpreted and reacted differently to specific words used in for some items. The younger participants, for example, may have understood the word “foolish” in the same sense meant by the researchers (i.e., “embarrassed”), but older students may have a different context for this word that affected how they responded (e.g., “dumb”). The fit statistics were better for the middle school students, which may indicate that wording for some items elicited different interpretations and responses from the two student groups. Wording and sentence structure that are age-appropriate are important in scale development, and further study with the WAGS should address this limitation.

A final limitation relates to the number of goals assessed by the WAGS and the extent to which the WAGS adequately assesses all achievement goals. Although the means for each achievement goal are consistent across Studies 1 and 2 (M_Mastery_ = 2.31 and 2.53; M_Performance−Approach_ = 1.78 and 1.86; M_Performance−Avoidance_ = 1.67 and 1.20 respectively), the means for each factor are relatively low, suggesting the possibility that students are responding to other goals that may affect their responses. Our results suggest that other goals, not measured here, may have been more influential with students than the mastery and performance goals explored in this study. This result, furthermore, is consonant with recent refinements in achievement goal research (e.g., Elliot et al., 2011), though more research is needed that examines the conditions in which alternative goals influence student writing. We have started a line of inquiry in this area. We have studied, for example, how *task completion* goals may become salient to students when they are stressed by academic deadlines, and we are examining how achievement goals evolve over an extended period of time (See also, Zhao et al., 2013). We believe that lines of inquiry such as these can clarify how students' writing goals are affected by contextual factors and can help researchers better identify the writing conditions and writing achievement goals most likely to develop confident, engaged writers.

## Ethics statement

This study was carried out in accordance with the recommendations of University of Nebraska-Lincoln IRB with written informed consent from all subjects. All subjects gave written informed consent in accordance with the Declaration of Helsinki. The protocol was approved by the University of Nebraska-Lincoln IRB.

## Author contributions

Substantial contributions to the conception or design of the work; or the acquisition, analysis, or interpretation of data for the work (MYS, MZ, RZ, RB, MD, and DK). Drafting the work or revising it critically for important intellectual content (MYS, MZ, RZ, RB, MD, and DK).

### Conflict of interest statement

The authors declare that the research was conducted in the absence of any commercial or financial relationships that could be construed as a potential conflict of interest.
